# Non-Enzymatic Assembly of a Minimized RNA Polymerase Ribozyme

**DOI:** 10.1002/syst.201900004

**Published:** 2019-02-20

**Authors:** Falk Wachowius, Philipp Holliger

**Affiliations:** a Medical Research Council Laboratory of Molecular Biology, Francis Crick Avenue, Cambridge, CB2 0QH (UK)

**Keywords:** nucleic acids, origin of life, ribozymes, RNA, templated ligation, phosphorimidazolide

## Abstract

Central to the “RNA world” hypothesis of the origin of life is the emergence of an RNA catalyst capable of RNA replication. However, possible replicase ribozymes are quite complex and were likely predated by simpler non-enzymatic replication reactions. The templated polymerisation of phosphorimidazolide (Imp) activated ribonucleotides currently appears as the most tractable route to both generate and replicate short RNA oligomer pools from which a replicase could emerge. Herein we demonstrate the rapid assembly of complex ribozymes from such Imp-activated RNA fragment pools. Specifically, we show assembly of a newly selected minimal RNA polymerase ribozyme variant (150 nt) by RNA templated ligation of 5’-2-methylimidazole-activated RNA oligomers <30 nucleotides long. Our results provide support for the possibility that complex RNA structures could have emerged from pools of activated RNA oligomers and outlines a path for the transition from non-enzymatic/chemical to enzymatic RNA replication.

The cornerstone of a hypothetical primodial RNA-dominated biology (an “RNA world”)^[1]^is an RNA molecule capable of replicating its own sequence and initiating Darwinian evolution. RNA polymerase ribozymes (RPR) generated by *in vitro* evolution^[2]^ are credible candidates for a modern-day reconstruction of such a RNA replicase. However, how such large and complex ribozymes could have emerged from the pools of short RNA oligomers accessible from prebiotic chemistry (<30 nts)^[3]^ is currently unclear. A plausible approach towards the synthesis of long, more complex RNAs involves their hierarchical assembly from short RNA oligomers by iterative ligation reactions. This approach has previously been exemplified enzymatically for the templated assembly of complex ribozymes from RNA oligomers (for the sunY self-splicing intron),^[4]^ a cross-chiral RNA ligase,^[5]^ a RPR by the hairpin ribozyme,^[6]^ or more recently from RNA oligomer and trinucleotide triphosphates.^[7]^ However, these approaches all suppose the pre-emergence of a ligase ribozyme activity to drive assembly. Here, we explore a non-enzymatic approach based on the widely used phosphorimidazolide (Imp) activation chemistry (Figure 1), which has been shown to enable *de novo* (non-templated)^[8]^ and templated RNA polymerisation^[3a,9]^, seeking to connect enzymatic (ribozyme-catalyzed) RNA polymerisation to the simpler non-enzymatic RNA polymerisation reactions that must have preceded it.^[10]^ We first defined optimal reaction conditions that would allow RNA polymerase ribozyme (RPR) activity to proceed in eutectic ice phases, where both *de novo* as well as templated non-enzymatic polymerisation of RNA could be demonstrated.^[8b,11]^

We could identify improved reaction conditions for in-ice RPR activity based on a switch of the buffer system and a slight increase of the pH (Figure S1, S2). Investigating pH vs. Mg^2+^ concentration ([Mg^2+^]) dependence (for MgCl_2_) of RPR activity in ice over a [Mg^2+^] range of 5–30 mM and a pH range of 8.3–10.5 revealed a trend towards enhanced activity at higher pH values with decreasing [Mg^2+^] (Figure S1, S2). As this trend is not transferable to ambient temperatures (Figure S1), it likely reflects ice-specific ion distribution between solid ice- and liquid eutectic phase. Subsequently this led to new reaction conditions (–7°C, 10 mM MgCl_2_, CHES pH 9) that resulted in a 20-fold drop in [Mg^2+^] required for optimal in-ice RPR activity (Figure S1) in comparison to the previously applied optimal reaction conditions for RPR polymerisation in ice (–7°C, 200 mM MgCl_2_, TRIS pH 8.3)^[7,12]^ reducing RNA degradation.

We further optimized RPR activity (while minimizing RPR size) using in–ice selection by CBT (Compartmentalized bead-tagging) *in vitro* evolution.^[2b,7]^ Starting from RPR variants Z, W, Y^[7]^ using different randomised linker lengths (12nt, 18nt, 24nt) connecting the RPR catalytic core to the accessory domain (Figure 2). After 8 rounds of CBT selection, only variants with a 12 nt linker region remained and among these we identified 3 clones (C1, C2, K) with improved activity on a new selection template (Figure S3). All three variants comprised four core mutations C67U, G95A, C119U and A145Cand therefore derived from the W variant pool.

In particular one clone (named K) showed improved RNA polymerase activity on the new primer/template combination in comparison to the best RPR variant (Z) and additionally could be shortened to 180 nt compared to Z (195 nt)^[2b]^ without loss of activity (Figure 2). The K ribozyme accessory domain could be truncated further^[13]^ resulting in an even shorter RPR variant (F) of only 150 nt, which retained robust polymerase activity but only in ice (Figure S4, 5) and in particular at higher RPR concentrations (7 μM), which had previously proved inhibitory. This results in improved polymerisation activity on some challenging templates (Figure S5). After having minimized RPR size substantially and optimized activity, we investigated if the F ribozyme could be assembled from the short, activated RNA oligomers, such as would be accessible from untemplated non-enzymatic RNA polymerisation reactions.^[14]^ Non-enzymatic polymerisation of RNA oligomers from 5’-phosphorimidazole-activated monomers in ice generates preferentially short oligomers with maximum lengths of ~20 nt with a mixed base composition.^[8b]^ On montmorillonite clay longer oligomers have been observed but only for low complexity sequences.^[15]^ Optimized templated non-enzymatic RNA replication using phosphorimidazole (or similar phosphate activation) chemistry is increasingly able to generate mixed sequence oligomers suggesting that mixed RNA oligomer sequence pools in the ≤30 nt size range may be prebiotically plausible. However as Imp activated monomers are preferentially introduced,^[3a]^ ligation of oligos is much less efficient^[9c]^, which hindered polymerisation of 5’-Imp activated RNA oligomers so far.

To test this conjecture, we first explored non-enzymatic assembly of 5’-phosphor-2-methyl-imidazolide (2-MeImp)–activated RNA eicosamers (20 nt) by dividing the class I ligase derived catalytic core of the RPR (100 nt) into five 20-mer segments. Upon addition of the complementary RNA splints (20 nt) and applying the same optimised reaction conditions identified for in-ice activity of the RPR (10 mM Mg^2+^, CHES, pH 9) the spontaneous assembly of the full length catalytic core sequence (Figure 3) with 5.4% yield (24 h, 37°C) could be observed.

Next, we sought to assemble a full length RPR (variant F) in the same manner. To this end the whole F RPR sequence was divided into four pieces, each comprising ~38 nt and their 5’ phosphate termini were activated with 2-MeImp. After addition of four complementary RNA splints (24 nt each), we again observed assembly of the whole functional F ribozyme sequence (24 h at 37°C) with a total yield of 1.7% (Figure S6). This shows that longer RNA oligomers, should they become available from non-enzymatic polymerisation reactions, can be assembled in analogous fashion despite their increased tendency for secondary structure formation.^[16]^

Finally, we sought to assemble the whole F RNA polymerase ribozyme from RNA oligomers short enough to be accessible from non-enzymatic polymerisation (≤30 nt). This requires the definition of six mutually exclusive (orthogonal) assembly sites in the RPR sequence, with a concomitant steep increase in the potential for mis-assembly. To this end we split the F ribozyme sequence of 150 nt into seven pieceswith arbitrary ligation sites (6×20 nt, 1×30 nt) and activated the 5’-phosphate termini with 2-MeImp. After the addition of the corresponding six complementary RNA splints (5×20 nt, 1×30 nt), we observed the ligation of the full-length ribozyme sequence (Figure 4). Correct assembly of full-length F ribozyme was verified by sequencing (SI Materials & Methods) and full RNA polymerase ribozyme activity was confirmed by primer extension in ice (of the recovered *in vitro* transcribed version, SI Materials & Methods) (Figure S7) using the optimized buffer conditions.

Our results show how complex RNA structures could have been generated non-enzymatically in a plausible prebiotic setting by the iterative ligation of multiple phosphorimidazole activated RNA oligomers. We provide a proof-of-principle demonstration for the spontaneous assembly of a functional 150 nt RNA polymerase ribozyme (*F_1234567_*) from up to 7 short (20–30 nt), non-functional 5’-2-methylimidazole-activated RNA oligonucleotides via splint assisted non-enzymatic ligation in a “one pot” reaction. The final yield of the full-length ribozyme from 7 pieces is currently relatively modest (0.5 %), which is likely related to the chemical instability and short half-life of the phosphorimidazole activating groups in aqueous solution, the incomplete formation of ligation junctions due to RNA misfolding and incomplete ligation of assembled junctions. Furthermore, the chemical reactivity of 5’-phosphorimidazole activated RNA oligonucleotides is in general reduced, compared to the equivalent activated RNA monomers, also related to the fact, that the latter is assisted by beneficial interactions of the imidazole leaving groups of the incoming monomer and the next downstream monomer^[17]^ that are absent in the oligonucleotide ligation approach. Reactivation of the 5’-phosphates with imidazole in combination with binding to a solid matrix and/or the introduction of a cycling regime, as demonstrated for both templated RNA monomer polymerisation^[3a,9d]^ as well as enzymatic ribozyme assembly^[6]^, may allow improved reaction yields. While enzymatic ligation strategies often require specific ligation sites that have to be selected (or engineered) prior to ligation, the here described non-enzymatic ligation approach shows a much more general ligation behaviour i. e. with no apparent dependency on the nucleotide identity at the ligation junctions.

The new minimised RNA polymerase ribozyme (F) (comprising only 150 nt) represents one of the shortest RPR, that shows robust RNA polymerase activity on difficult template sequences in the preferential medium of water-ice at–7°C. This could pave the way for future RPR catalysed RNA self-replication, based on a combination of RNA oligomer synthesis by enzymatic NTP polymerisation and non-enzymatic templated ligation of these oligonucleotides based on phosphorimidazolides at ambient temperatures by applying the same overall reaction conditions (10 mM Mg^2+^, CHES pH 9), while the substantial decrease in Mg^2+^ concentrations are beneficial regarding both RNA degradation and dsRNA strand separation.

The first primitive genetic systems likely propagated themselves by non-enzymatic RNA replication initiated from pools of short oligomer substrates provided by prebiotic chemistry^[8b,15]^ until more efficient RNA replicase ribozymes emerged at a later stage.^[18]^ Hence, assembly of short RNA oligomer into longer strands would have been a critical transition in boosting pool complexity allowing more efficient enzymatic replicators to emerge. These may have initially similarly utilized oligomer substrates before transition into the more abundant short tetra-, tri- and dinucleotide and eventually mononucleotide substrates^[2d]^, i. e. RNA replicators increased in size and complexity, while their substrates decreased in size.^[19]^ The here explored combination of enzymatic RNA polymerisation and non-enzymatic RNA ligation based on 2-methylimidazolide activated oligonucleotides thus represents a proof-of-principle for a plausible step in a transition scenario from prebiotic oligomer pools to RNA self-replication. Nevertheless, it remains possible and even plausible that different enzymatic and non-enzymatic RNA polymerisation-/ligation-based replication systems might have operated in parallel, coexisting by exploiting orthogonal substrate pools and competing for resources, dependent on substrate availability and environmental conditions.

## Supplementary Material

Supporting information for this article is available on the WWW under https://doi.org/10.1002/syst.201900004

Supp Info

## Figures and Tables

**Figure 1:**
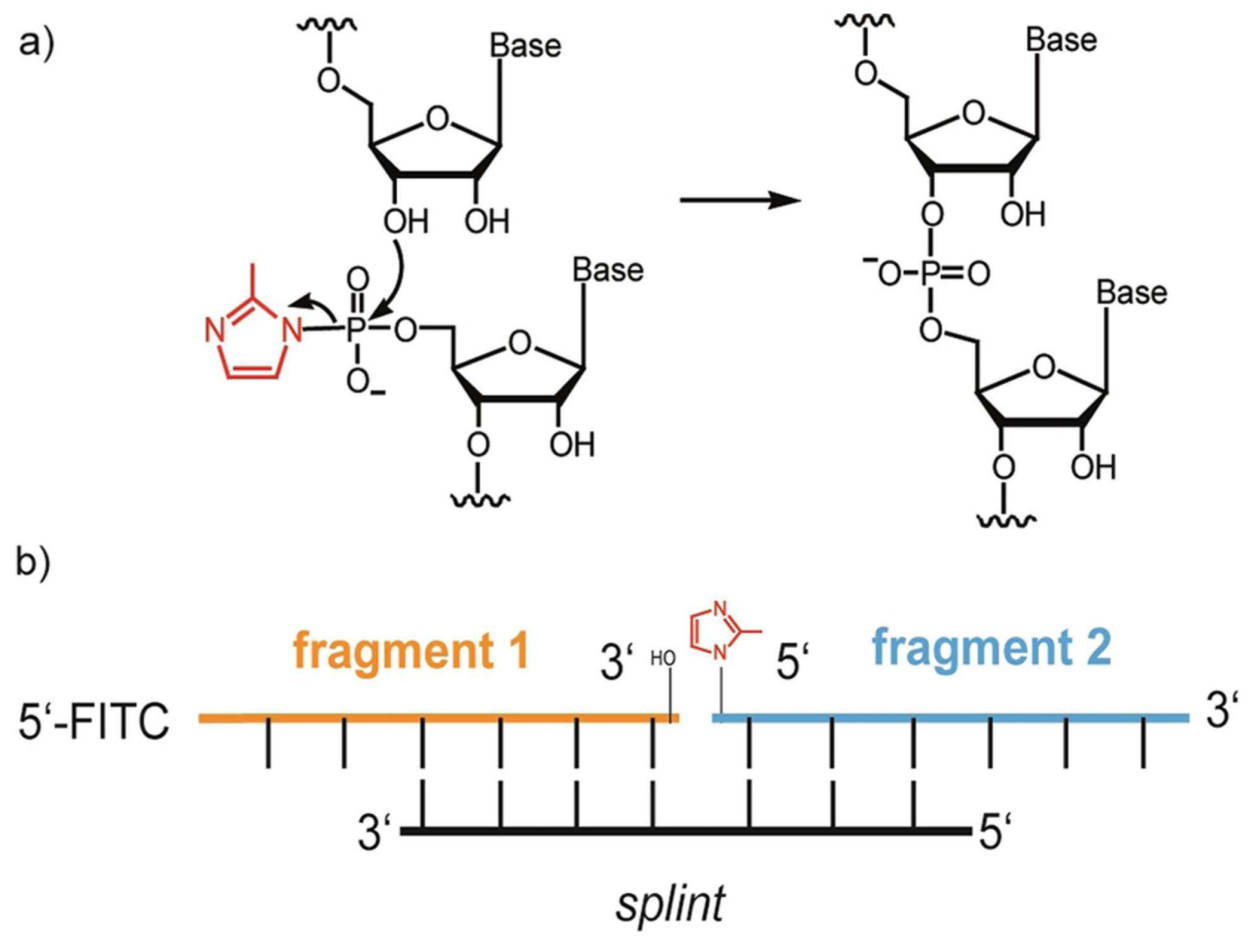
RNA ligation by phosphoimidazolide (Imp) activation chemistry. a) Nucleophilic attack of the 3’-OH on an activated Imp-activated 5’-phosphate (2-methyl-imidazole (red)) results in phosphodiester bond formation. b) Ligation scheme comprising upstream (blue) and Imp–activated downstream RNA fragment (orange) and RNA splint oligonucleotide, that positions the ligation fragments by Watson–Crick base pairing.

**Figure 2:**
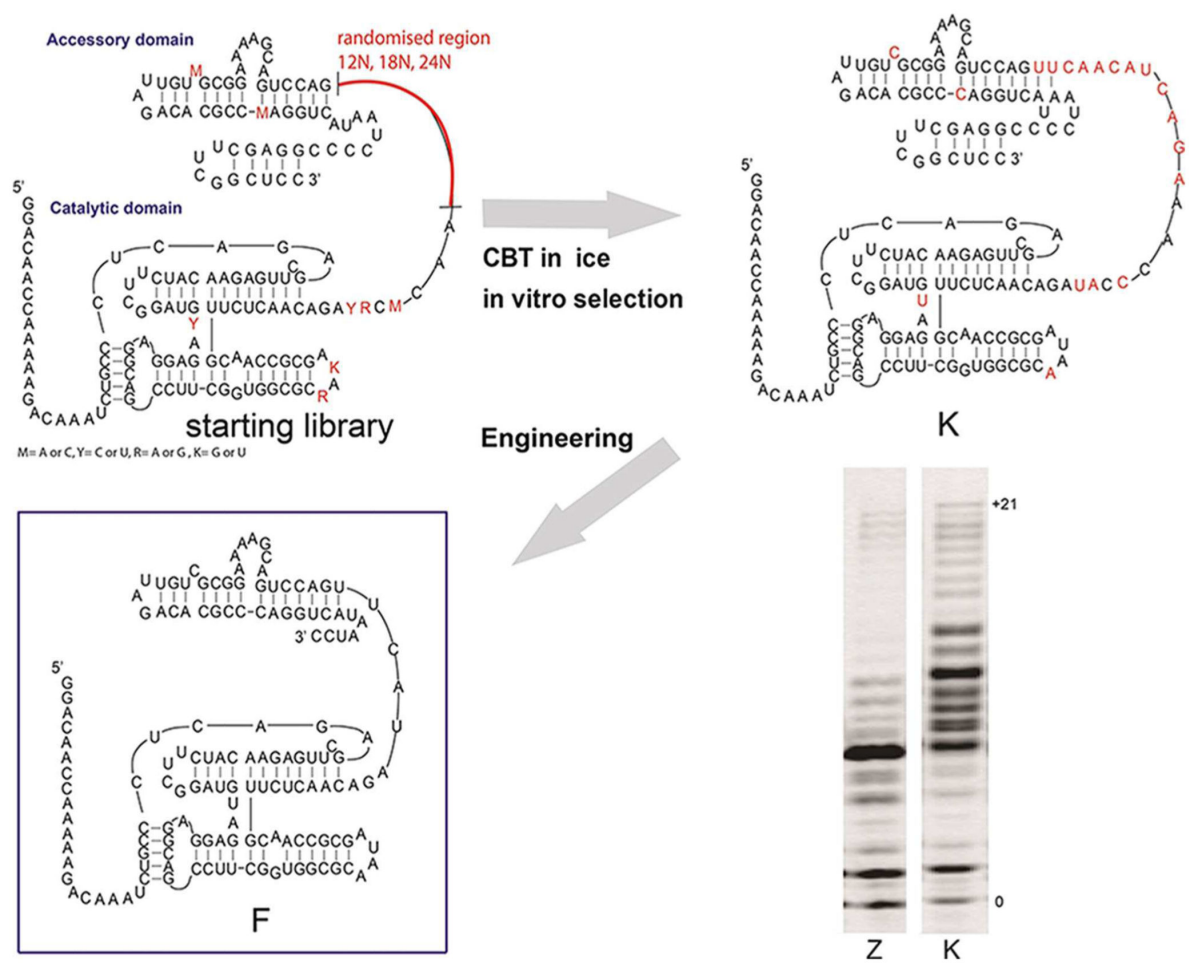
Generation of the F RNA polymerase ribozyme. In-ice selection of different linker domain lengths (12–24 N) and defined mutations (red) resulted in new K RPR with improved activity compared to the Z RPR (bottom right panel). The K linker and accessory domain could be further truncated without loss of activity to yield the minimized F RPR.

**Figure 3:**
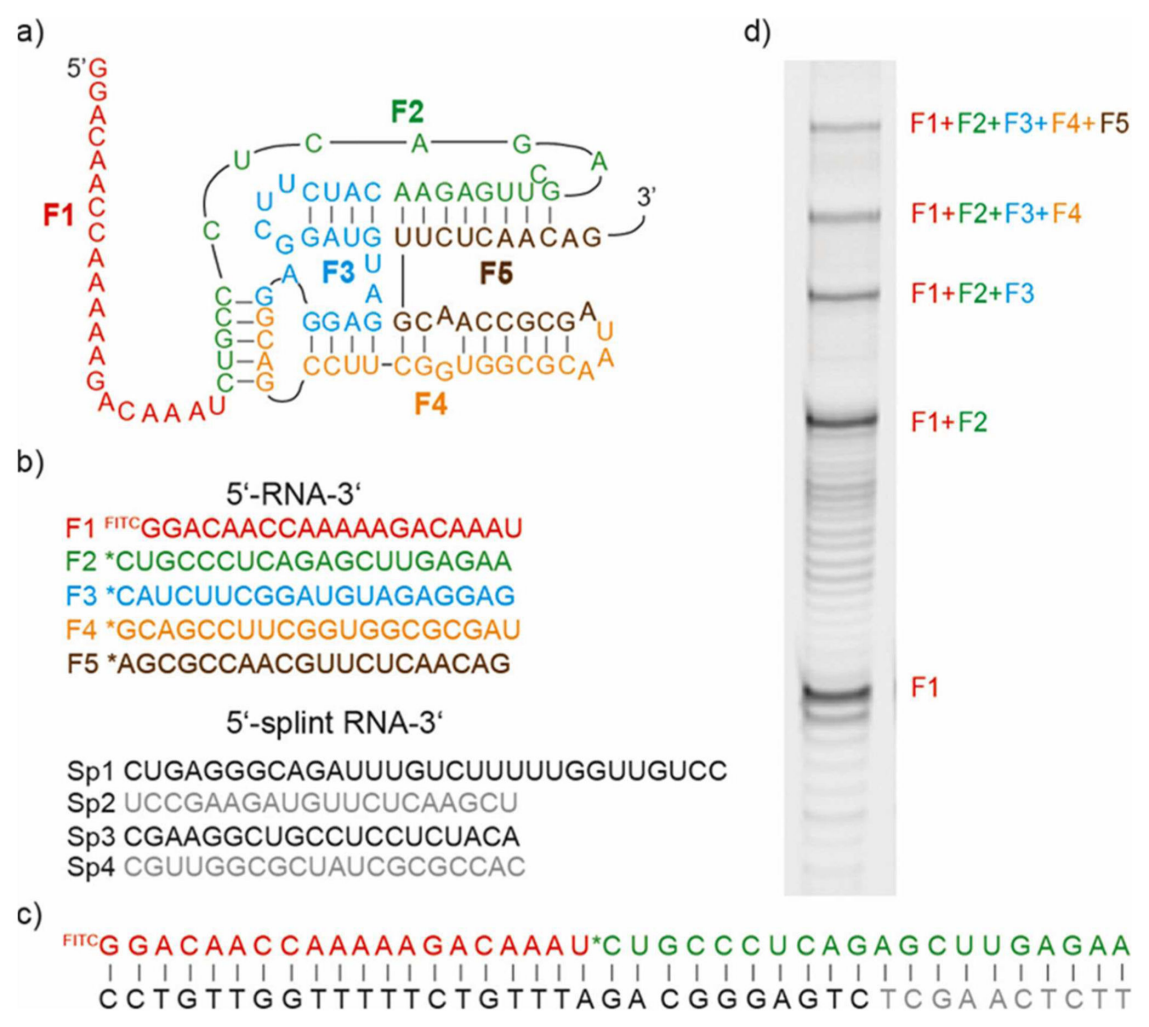
Non-enzymatic templated ligation of the F ribozyme catalytic core. a) Secondary structure of the catalytic core; each RNA fragment (20 nt) is colour coded. b) Primary sequence of the RNA fragments (F1-F5), color coded as in (a) and the corresponding RNA splints (Sp1-Sp4),*5’-phosphor-2-methylimidazole, FITC: Fluorescein isothiocyanate. c) Templated ligation: 5’-2-MeImp activated RNA fragments (F1+F2) base pair with their corresponding splint oligos (Sp1+Sp2). d) PAGE gel of the non-enzymatic assembly of the catalytic core (24 h). Band intensities (mean of three individual experiments): F_1_: 39%, F_12_: 34%, F_123_: 13.3%, F_1234_: 8.3%, F_12345_: 5.4%.

**Figure 4:**
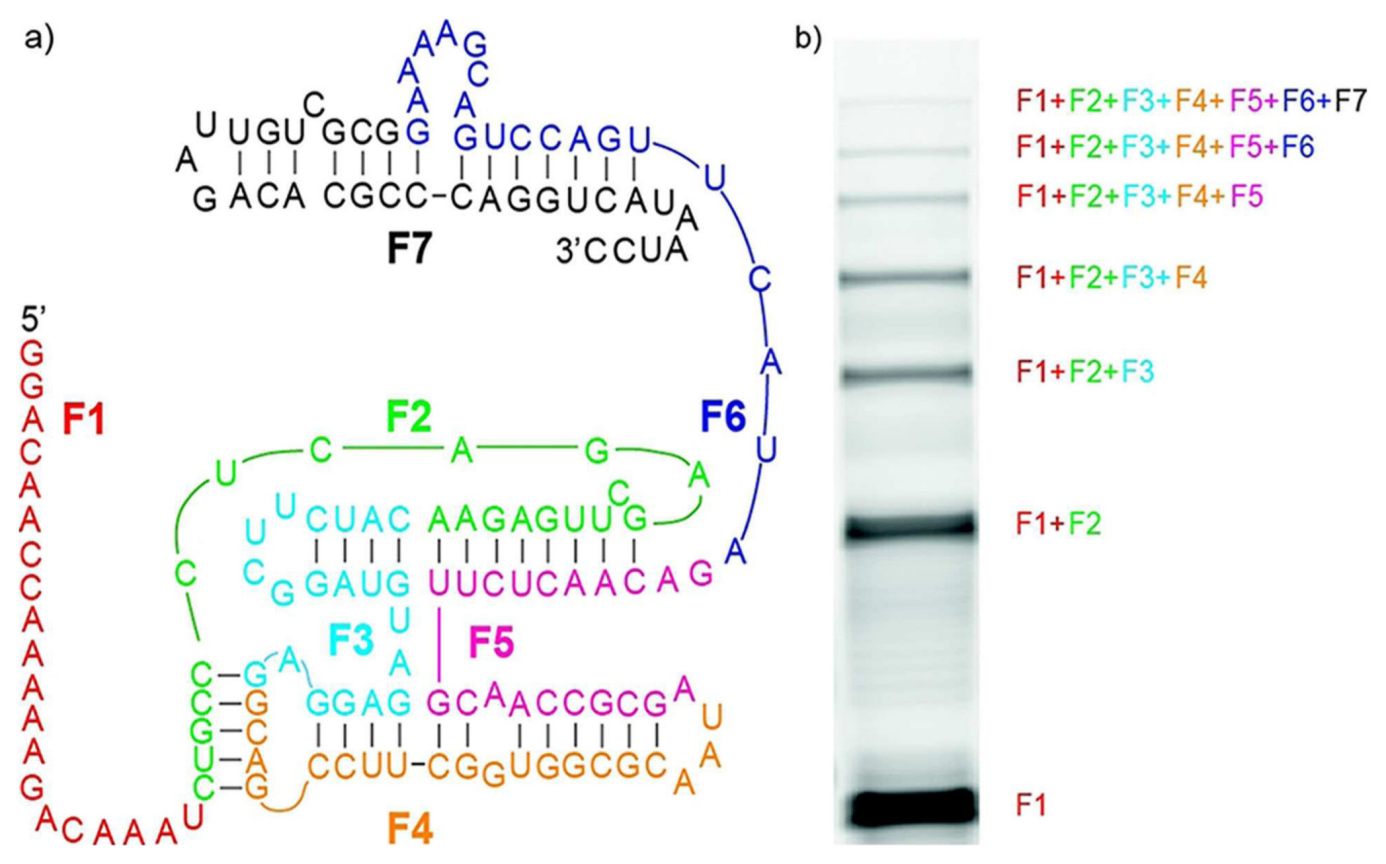
Functional RPR assembly from short RNA fragments. a) Secondary structure of the F ribozyme. Different colours indicate different fragments (F1-7). b) PAGE gel of non-enzymatic assembly reaction showing full length assembly band (F_1234567_: F1+F2+F3+F4+F5+F6+F7).
