# Supplementary material for: Non-Enzymatic Assembly of a Minimized RNA Polymerase Ribozyme
Source: ChemSystemsChem. Author manuscript; Available in PMC 2019 Oct 31. (PMC6823052; doi:10.1002/syst.201900004)
Supplement: Supp Info [file EMS84580-supplement-Supp_Info.pdf]

Supporting Information

© Copyright Wiley-VCH Verlag GmbH & Co. KGaA, 69451 Weinheim, 2019

Non-Enzymatic Assembly of a Minimized RNA Polymerase Ribozyme

Falk Wachowius and Philipp Holliger*© 2019 The Authors. Published by Wiley-VCH Verlag GmbH & Co. KGaA.
This is an open access article under the terms of the Creative Commons Attribution License, which permits use, distribution and reproduction in any medium, provided the original work is properly cited.

Table of Contents

Supplementary Data

Materials & Methods 3
1. Chemicals 3
2. Oligonucleotides 3
3. Compartmentalized bead-tagging (CBT) 3
4. Synthesis of 5'phosphorimidazole RNA oligonucleotides 4
5. Templated RNA ligation 4
6. Functional activity and sequencing of full-length ligation products 4
6.1 Sequencing result of full-length ligation product F 1-4 4
6.2 Sequencing result of full-length ligation product F 1-7 5
7. RPR catalysed primer extensions 5
Supporting figures: 6
Figure S1. Optimisation of in-ice RNA polymerase ribozyme activity. 6
Figure S2. Optimisation of in-ice RNA polymerase ribozyme activity II. 7
Figure S3. Selection library. 8
Figure S4. Selected polymerase ribozyme clones. 9
Figure S5. In-ice ribozyme activity. 10
Figure S6. Non-enzymatic RNA polymerase ribozyme assembly. 11
Figure S7. Activity of assembled ribozyme. 12
Supporting Tables 13
Table S1. Table of DNA and RNA sequences. 13
Supplemental References 18

Materials & Methods

1. Chemicals

2-Methylimidazole, N-(3-Dimethylaminopropyl)-N′-ethylcarbodiimide hydrochloride (EDC) and reaction buffers Tris(hydroxymethyl)aminomethan (TRIS), *N,N*-Bis(2-hydroxyethyl)-2-aminoethanesulfonic acid (BES), 3-[4-(2-Hydroxyethyl)piperazin-1-yl]propane-1-sulfonic acid (EPPS) and2-(Cyclohexylamino)ethanesulfonic acid (CHES) were from Sigma Aldrich. NTP's for RPR catalysed primer extensions were ordered as lithium salts from Roche.

2. Oligonucleotides

Table S1 specifies the DNA and RNA oligonucleotides used in this study. All DNA and RNA oligonucleotides were purchased from Integrated DNA Technologies (IDT). DNA templates for RNA in-vitro transcription were generated by fill-in of two DNA oligonucleotides (in verse template sequence and primer P T7) using GoTaq (Promega) followed by DNA purification using QiaQuick (Qiagen). RNAs were transcribed using the MegaShortScript high-yield transcription kit (Ambion) and purified by RNeasy (Qiagen). Oligonucleotides were purified, if necessary, by denaturing PAGE on 10%, 15%, or 20% denaturing polyacrylamide gels containing 8 M urea. After visualisation by UV shadowing, oligonucleotides were excised from the gel, extracted overnight by crush and soak into 0.3 M NaOAc, followed by precipitation with absolute ethanol on dry ice, two washing steps with 70% ethanol anddried by speed vac. The oligonucleotide concentration was determined by Nanodrop (Thermo Fisher) at a wavelength of 260 nm.

3. Compartmentalized bead-tagging (CBT)

Compartmentalized bead-tagging (CBT) selection procedure[1] with the primer extension step performed in ice.[2] The CBT selection strategy is described in detail in previous papers.[1-2] In short, biotinylated ribozyme genes are bound to streptavidin-coated magnetic beads (MyOne Streptavidin C1 Dynabeads, Invitrogen) (**1**) and clonal ribozyme populations are produced by coupled in-emulsion transcription (Megashortscript High Yield transcription kit, Ambion) (**2**), followed by decoration of the beads with primer/template duplexes (**3**). After in emulsion RPR catalysed primer extensions in the eutectic-phase of water-ice at -7°C with 200 mM $MgCl_2$, 50 mM TRIS pH 8.3 (**4**), extended primers are selectively amplified by rolling circle amplification (RCA) based on predefined DNA minicircles (DC +7 RP, DC 0 RP, DC -3 RP and DC +6 RP, DC +4 RP, DC 0 RP, DC -4 RP) (**5**). After staining with SYBR® Gold (**6**) active RNA sequences are isolated by FACS sorting (Sony Synergy) applying two different sorting gates(high gate: top 1-5 % of single beads, low gate: top 5-10 % of single beads, than mixed in a 1:1 ratio), (**7**) and amplified by PCR (30-38 cycles, depending on number of sorted single beads, 95˚C for 30 s, 50˚C for 30 s, 72˚C for 60 s) (**8**). Every second selection round an additional error prone PCR step (25 cycles, 95˚C for 30 s, 50˚C for 30 s, 72˚C for 60 s) using the GeneMorph II Random Mutagenesis Kit (Agilent) was introduced.

4. Synthesis of 5'phosphorimidazole RNA oligonucleotides

5'-phosphorylated RNA oligonucleotides (IDT) were suspended in Millipore water to a concentration of 100 µM. 20 µl were added to 20 µl of 1 M 2-Methylimidazole (pH 6) and 5.7 mg of EDC and the mixture was incubated for 2h at 25°C. The activated RNA oligonucleotides were purified and concentrated using Vivaspin® 500 concentrators, 3000MWCO (Sartorius) and, after concentration measurement by Nanodrop, directly used in the templated ligation reaction.

5. Templated RNA ligation

Templated RNA ligation reactions were performed in 10 µl volumes with 5' fluorescently labelled primer (0.5 µM),5' 2-Methylimidazole activated RNA oligonucleotides (1.5 µM each), RNA or DNA template strands (50 µM each), 10 mM $MgCl_2$ and 50 mM CHES pH 9.The reactions were incubated for 24h at 37°C, stopped by the addition of equal volumes of 10 mM EDTA in 8 M urea, 0.05% bromophenol blue and full-length ligation products were isolated on 20% polyacrylamide/ 8 M urea gels, analysed using a Typhoon Trio scanner (GE Healthcare) and quantified using Image Quant software (Molecular Dynamics).

6. Functional activity and sequencing of full-length ligation products

Full-length ligation products were excised from the gel, extracted as described above and reverse transcribed and amplified by PCR using primers (P F and P F rev) and the SuperScript® III One-Step RT-PCR System with Platinum® *Taq* DNA Polymerase (Invitrogen). After in-vitro transcription RNA sequences were analysed for their primer extension capability (S6, S7). For sequencing, DNA products were inserted into pGEM®-T (Promega) vectors and sequenced (Sanger sequencing) by Genewiz.

6.1 Sequencing result of full-length ligation product F 1-4, see Fig. S6, red marked nucleotides indicate the DNA sequence of the F ribozyme.

NNNNNNNNNGGGCCCCTCCCCCNCCGGGCGACCCTGCAGGCGGCCGCACTAGTGATTGATCGATC
TCGCCCGCGAAATTAATACGACTCACTATA**GGACAACCAAAAAGACAAATCTGCCCTCAGAGCTTG**
AGAACATCTTCGGATGTAGAGGAGGCAGCCTTCGGTGGCGCAATAGCGCCAACGTTCTCAACAGA
TACTTGACCTGACGAAAAGGCGCTGTTAGACACGCCCAGGTCATAATCCAATCCCGCGGCCATGG
CGGCCGGGAGCATGCGACGTCGGGCCCAATTCGCCCTATAGTGAGTCGTATTACAATTCACTGGCC
GTCGTTTTACAACGTCGTGACTGGGAAAACCCTGGCGTTACCCAACTTAATCGCCTTGCAGCACATC
CCCCTTTCGCCAGCTGGCGTAATAGCGAAGAGGCCCGCACCGATCGCCCTTCCCAACAGTTGCGCA
GCCTGAATGGCGAATGGACGCGCCCTGTAGCGGCGCATTAAGCGCGGCGGGTGTGGTGGTTACGC
GCAGCGTGACCGCTACACTTGCCAGCGCCCTAGCGCCCGCTCCTTTCGCTTTCTTCCCTTCCTTTCT
CGCCACGCTCGCCGGCTCTCCCCGTCAAGCTCTCAATCGGGGGCTCCCTTTAGGGCTCCCATTCAC
CGCTNTACGGCACCTCGACCCCACACAACTTGNNTACGGTGATGGTTCACGTACTGTGCCATCCTCC
CGCTCCACGCTTTTTCGCCCTCTGACTCCCCACTCTCTTCCNTNTATCTTCGCACTCTTGTTGCTACA
TGCAACTTTATGCCATCCCATTTTNGACTCTATTTCATCGACTTTTA

6.2 Sequencing result of full-length ligation product F 1-7, see Fig. S7, red marked nucleotides indicate the DNA sequence of the F ribozyme.

NNCATATGGTCGACCTGCAGGCGGCCGCACTAGTGATTGATCGATCTCGCCCGCGAAATTAATACGA
CTCACTATA**GGACAACCAAAAAGACAAATCTGCCCTCAGAGCTTGAGAACATCTTCGGATGTAGAG**
GAGGCAGCCTTCGGTGGCGCAATAGCGCCAACGTTCTCAACAGATACTTGACCTGACGAAAAGGC
GCTGTTAGACACGCCCAGGTCATAATCCAATCCCGCGGCCATGGCGGCCGGGAGCATGCGACGTC
GGGCCCAATTCGCCCTATAGTGAGTCGTATTACAATTCACTGGCCGTCGTTTTACAACGTCGTGACT
GGGAAAACCCTGGCGTTACCCAACTTAATCGCCTTGCAGCACATCCCCCTTTCGCCAGCTGGCGTAA
TAGCGAAGAGGCCCGCACCGATCGCCCTTCCCAACAGTTGCGCAGCCTGAATGGCGAATGGACGC
GCCCTGTAGCGGCGCATTAAGCGCGGCGGGTGTGGTGGTTACGCGCAGCGTGACCGCTACACTTG
CCAGCGCCCTAGCGCCCGCTCCTTTCGCTTTCTTCCCTTCCTTTCTCGCCACGTTCGCCGGCTTTCC
CCGTCAAGCTCTAAATCGGGGGCTCCCTTTAGGGTTCCGATTTAGTGCTTTACGGCACCTCGACCCC
AAAAAACTTGATTAGGGTGATGGTTCACGTAGTGGGCCATCGCCCTGATAGACGGTTTTTCGCCCTT
TGACGTTGGAGTCCACGTTCTTTAATAGTGGACTCTTGTTCCAAACTGGAACAACACTCAACCCTATC
TCGGTCTATTCTTTTGATTTATAAGGGATTTTGCCGATTTCGGCCTATTGGTTAAAAAATGAGCTGATT
TAACAAAAATTTAACGCGAATTTTAACAAAATATTAACGCTTACAATTTCCTGATGCGGTATTTTCTCCT
TACGCATCTGTGCGGTATTTCACACCGCATCAGGNGNCACTTTTCGGGGAAATGTGCGCGGAACCCT
NATTTGTTTATTTTTCTTAATACTTTNAAACATGGACTCGTCCATGATATAATAACCTTGATAATTGCCT
CACTTATATTGAAACAAGCACTATTATTATTATTCCATTCTTTCGGTGTTGCCTTGTTATCTATGTTTGC
GACATTTGCCTTCCCGGTTTTGC

7. RPR catalysed primer extensions

Primer extension reactions were performed in 10 µl volumes with 5' fluorescently labelled primer (0.5 µM), template (0.6 µM) and ribozme (0.7 µM). Primer/template and ribozyme were separately annealed (80°C, 2 min, 0.1°C/s to 17°C, 17°C for 10 min). After the addition of reaction buffer (final conc. 10 mM $MgCl_2$, 0.8 mM NTP's, 50 mM CHES pH 9 or other buffer/pH) or (final conc. 200 mM $MgCl_2$, 4 mM NTPs, 50 mM TRIS pH 8.3), the reaction mixture was flash-frozen on dry ice and transferred to a cooling bath (Grant) at -7°C or directly put to 17°C.The reactions were quenched by the addition of equal volumes of10 mM EDTA in 8 M urea, 0.05% bromophenol blue, 3 µM competing RNA oligonucleotide or.50 µM competing DNA oligonucleotide respectively. Reactions containing 200 mM $MgCl_2$ were stopped using quencing buffer containing 200 mM EDTA. Primer extensions were separated on polyacrylamide/8M urea gels and analysed using a Typhoon Trio scanner (GE Healthcare).

Supporting figures:

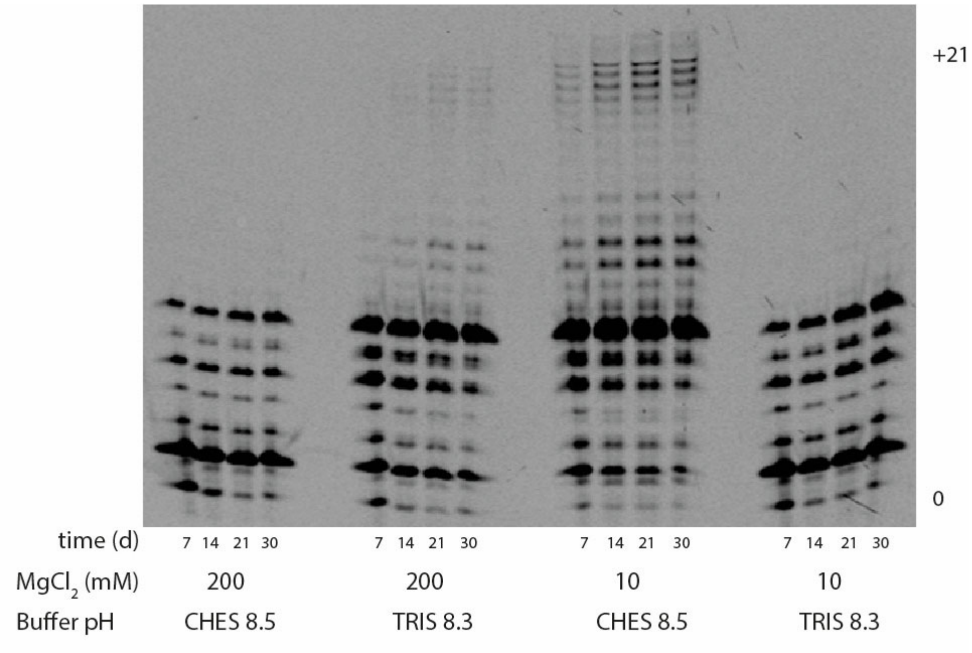

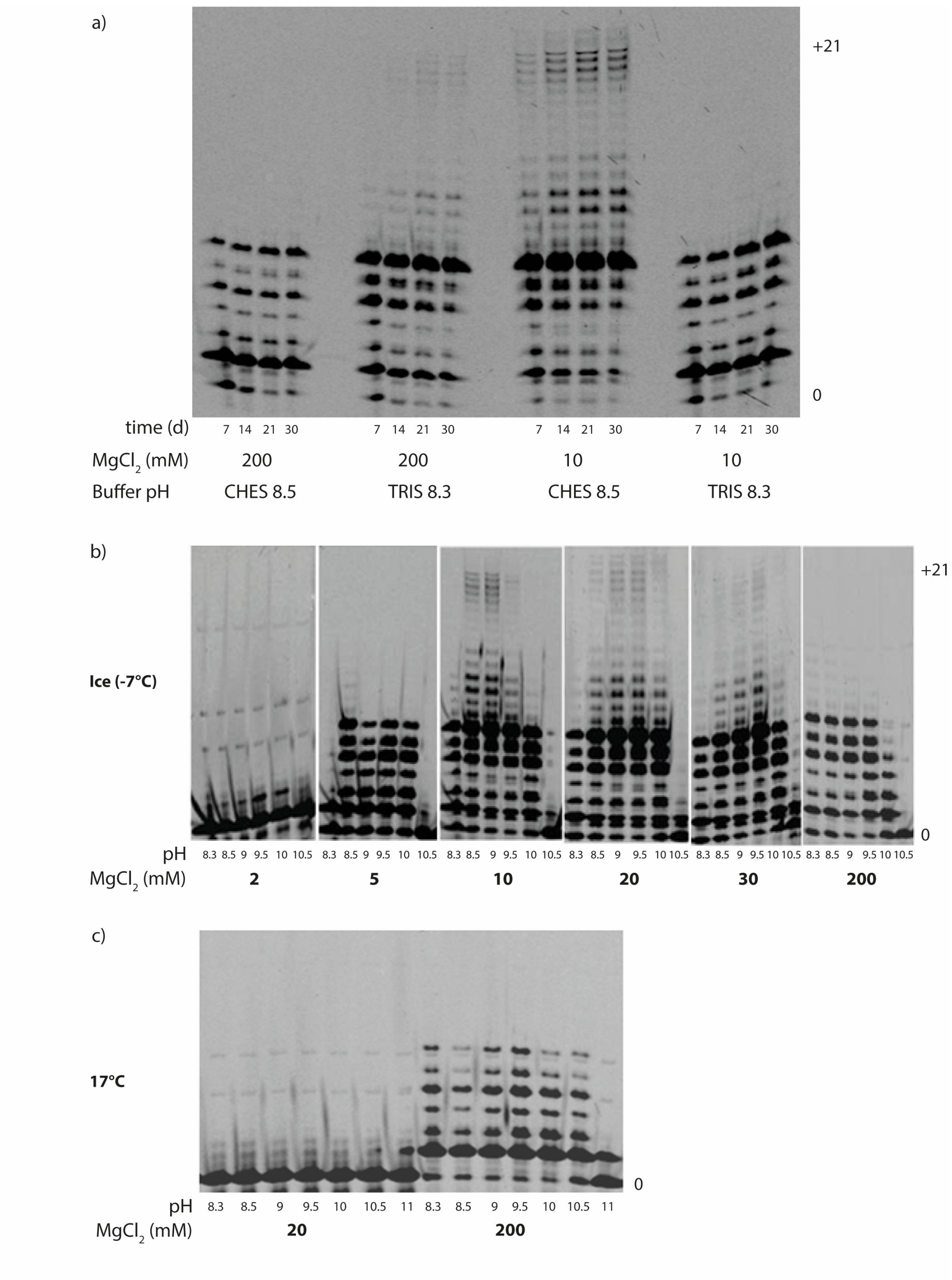

Figure S1. Optimisation of in-ice RNA polymerase ribozyme activity. a) Full reaction time course (30 d) of the RPR (Z) catalysed primer (P HP) extension along template (T HP) in the eutectic phase of water ice (-7°C) at 10 mM and 200 mM $MgCl_2$ applying CHES 8.5 or TRIS 8.3. b) PH dependence (pH 8.3-11) of the RPR (Z) catalysed extension on primer/template (P HP/T HP) in ice at -7°C (10d) using different $MgCl_2$ conc. (2 mM, 5 mM, 10 mM, 20 mM, 30 mM, 200 mM) (above) and c) at 17°C (4d) applying two different $MgCl_2$ concentrations (20 mM, 200 mM).

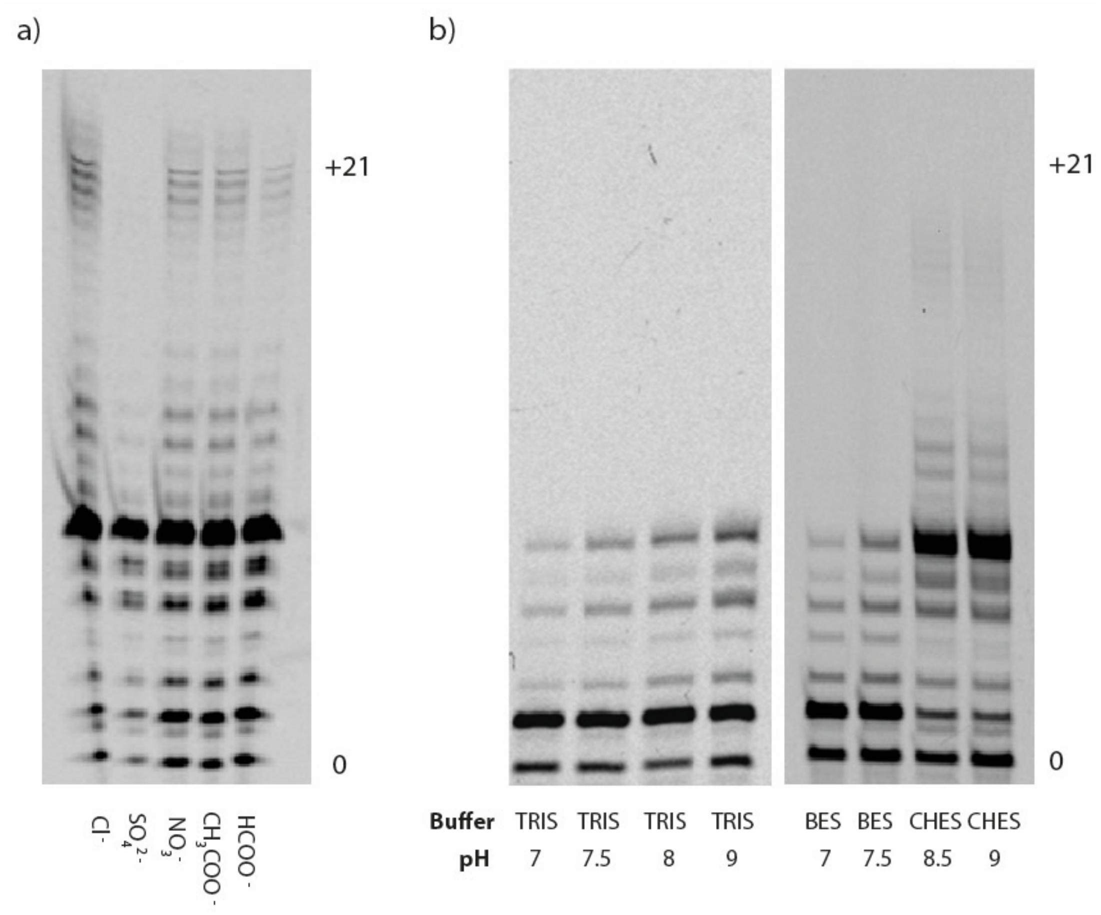

Figure S2. Optimisation of in-ice RNA polymerase ribozyme activity II. RPR (Z) catalysed primer (P HP) extension on template (T HP) in ice (-7°C) applying a) different Mg salts (Cl^-: Chloride, SO_4^{2-}: Sulfate, NO_3^-: Nitrate, CH_3COO^-: Acetate, $HCOO^-$: Formiate) with a concentration of 10 mM, at CHES pH 9 for 14 d, or b) different buffer systems (TRIS, BES, CHES) in the pH range 7-9 using a constant $MgCl_2$ concentration of 10 mM for 7d.

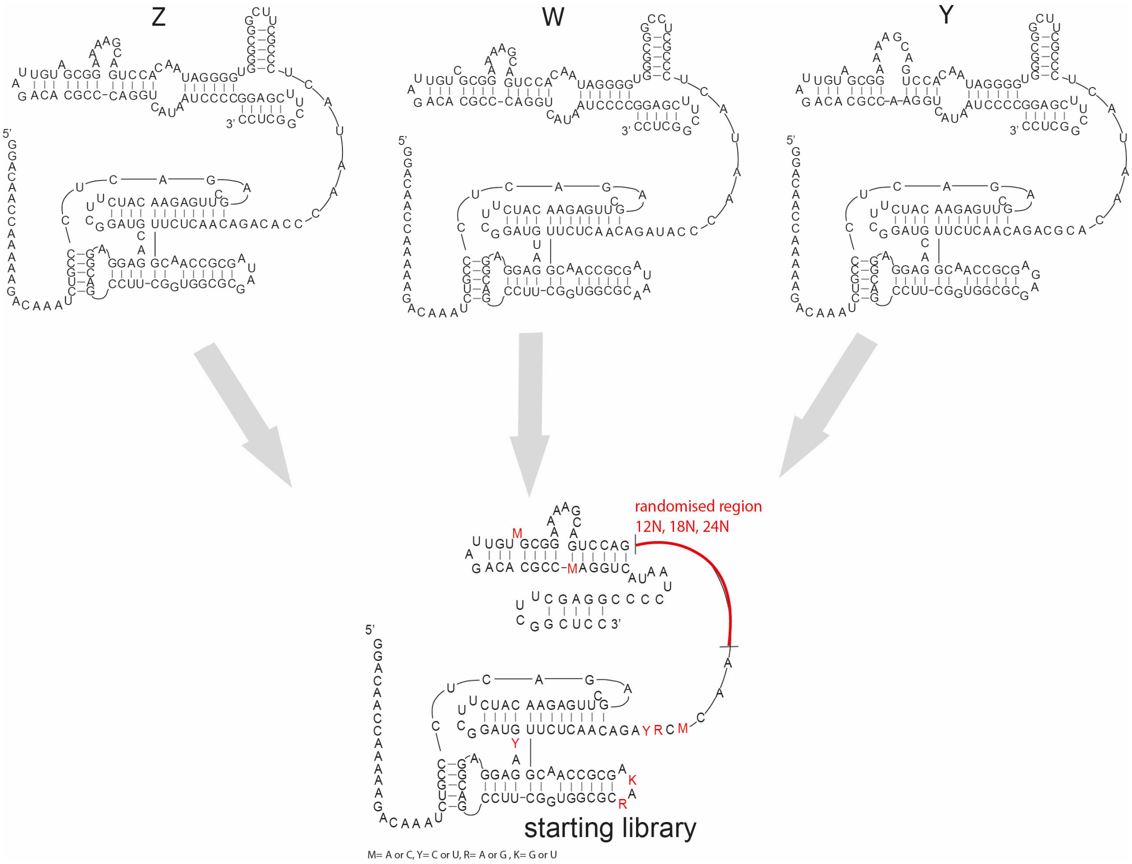

Figure S3. Selection library. The partly randomised starting library for selection was generated from three previously selected RPR variants (Z, W, Y) with a distribution of 50% for Z and 25% for W and Y and each comprising a randomised linker region of 12, 18 and 24 nt. The diversity of the library was increased by 2 rounds of error prone PCR (25 cycles, 95˚C for 30 s, 50˚C for 30 s, 72˚C for 60 s) using the GeneMorph II Random Mutagenesis Kit (Agilent). Red marked parts in the starting library indicate fully or partly randomised nucleotides/regions.

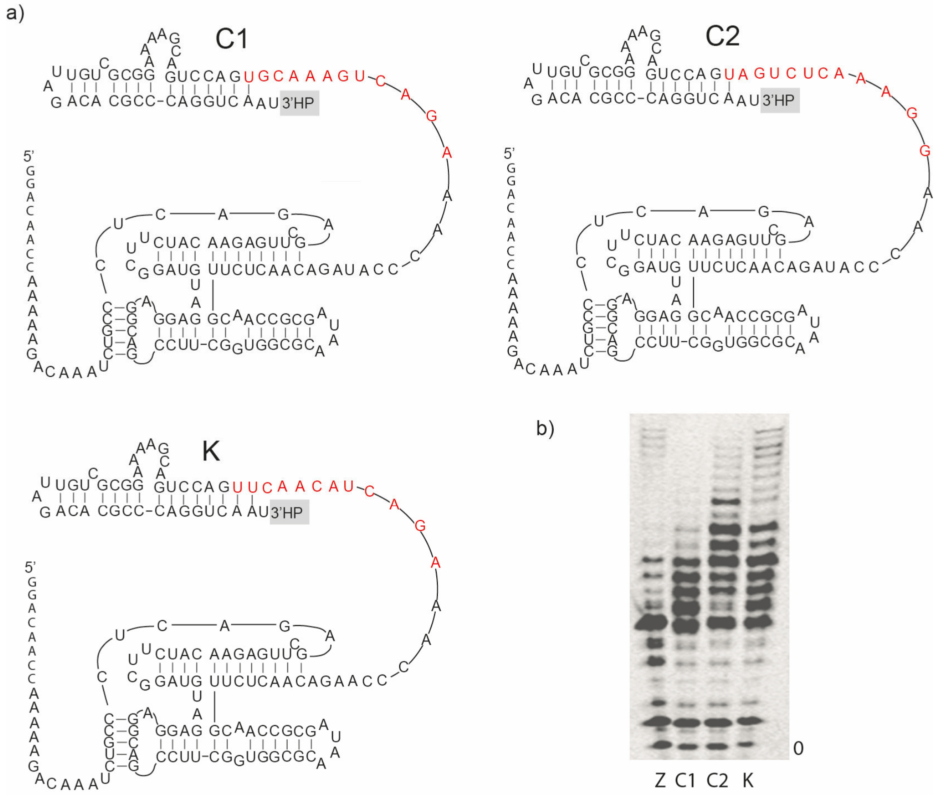

Figure S4. Selected polymerase ribozyme clones. a) Secondary structure of three different clones (C1, C2, K) identified by CBT selection, showing b) improved primer extensions on the selected primer/template combination (P HP/T HP) in comparison to the RPR variant Z; reaction conditions: ice (-7°C), 10 mM $MgCl_2$, CHES pH 9, 5d.

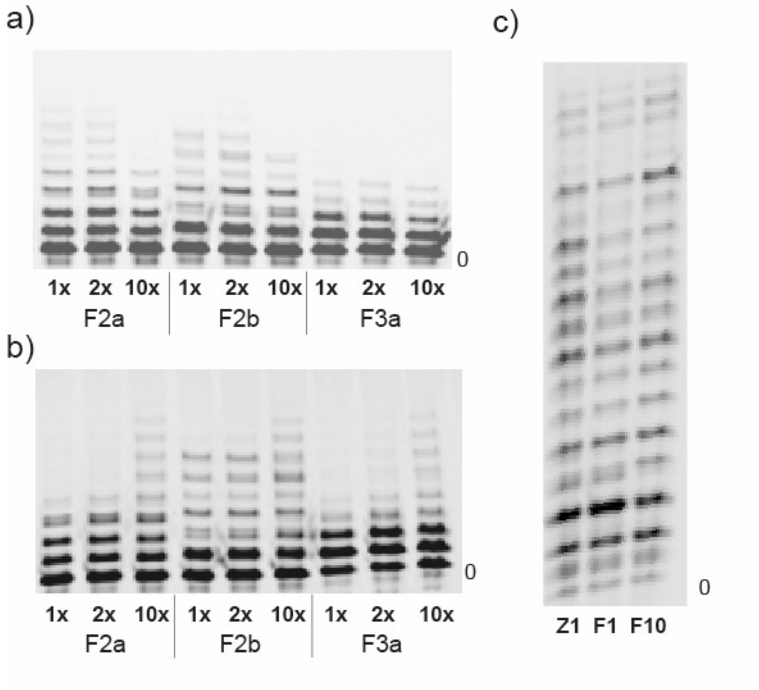

Figure S5. In-ice ribozyme activity. RPR concentration dependence of reactions in ice. PAGE gels of a) Z and b) F catalysed primer (P RP) extensions in ice (7d), on three difficult templates (F2a, F2b, F3a)encoding parts of the RPR structure applying different final ribozyme concentrations (1x: 0.7μM, 2x: 1.4μM, 10x: 7μM) and c) Z (Z1: 0.7μM) and F (F1: 0.7μM, F10: 7μM) primer (P RP) extension on an established template sequence (T RP).

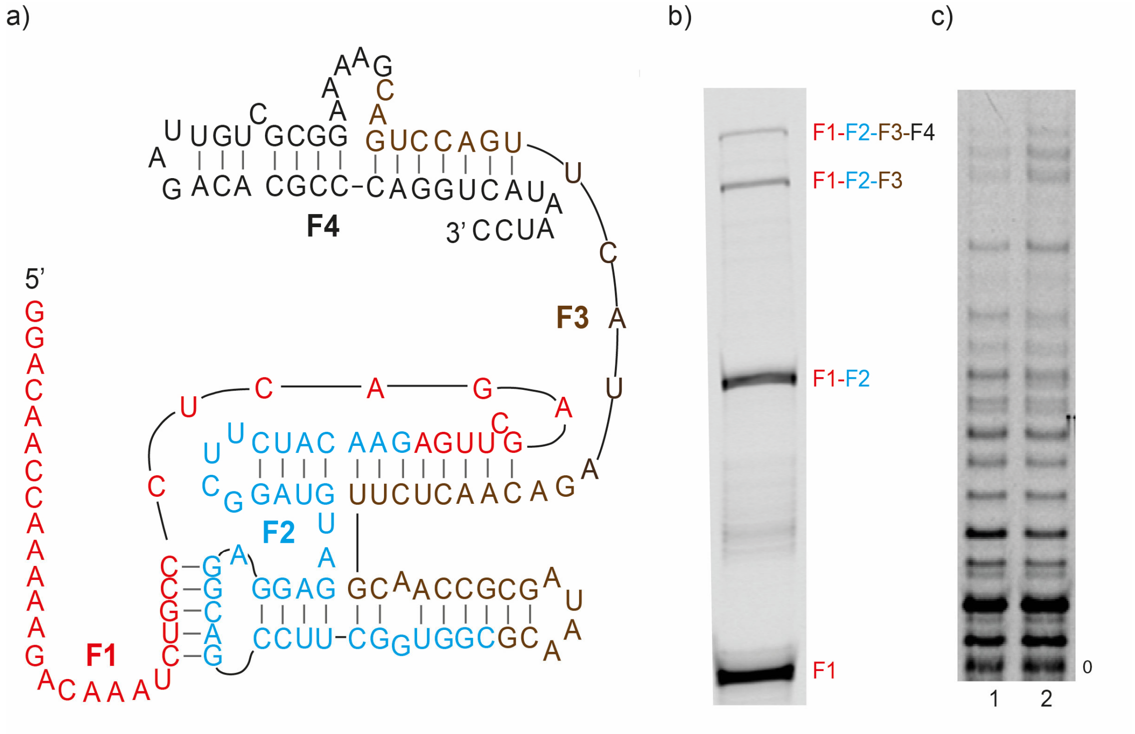

Figure S6. Non-enzymatic RNA polymerase ribozyme assembly. Templated non-enzymatic ligation of the full-length F RNA polymerase ribozyme (150 nt) from 4 pieces. a) Secondary structure of the F ribozyme; each ligation fragment (F1-F4) is indicated by a different color. b) PAGE gel of the non-enzymatic ligation of the full-length F ribozyme (F1+F2+F3+F4) after 24h.Quantification of band intensities yielded: F_1: 71.4%, F_{12} (F1 + F2): 22.7%, F_{123} (F1 + F2 + F3): 4.2%, F_{1234} (F1 + F2 + F3+ F4):1.7%. c) F catalysed primer (P RP)extension on template(T RP) in ice for 7d of 1) control F ribozyme and 2) full length-ligation variant(F_{1234}).

a)

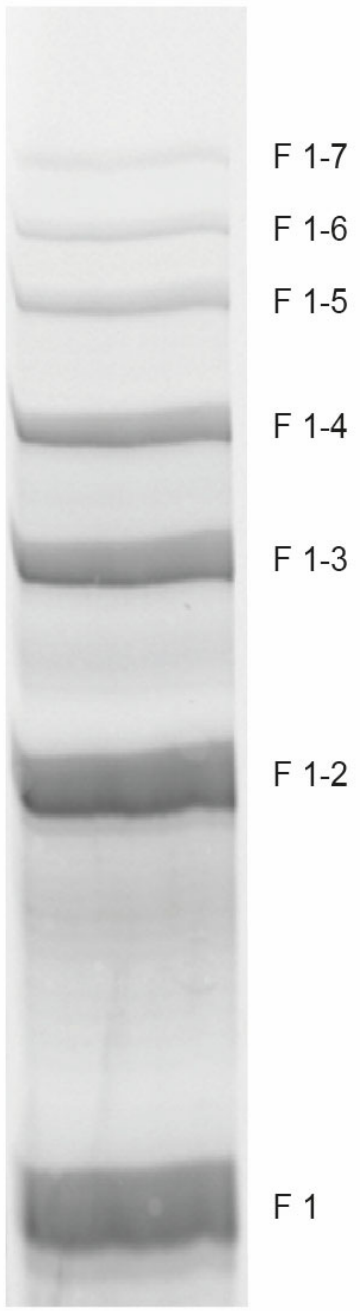

b)

	%
F 1-7	0.5
F 1-6	1.3
F 1-5	2.7
F 1-4	8.8
F 1-3	16.7
F 1-2	32.8
F 1	37.2

c)

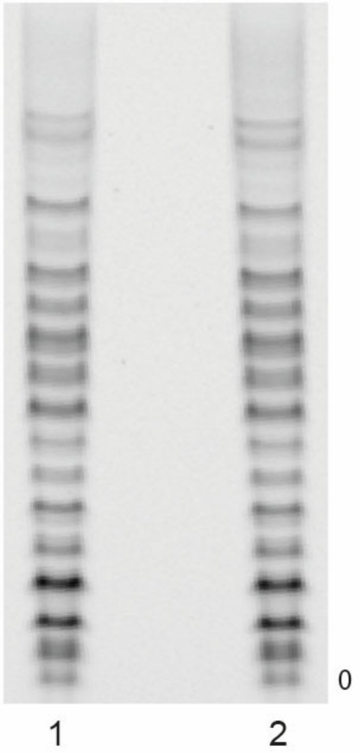

Figure S7. Activity of assembled ribozyme. a) PAGE gel of the templated non-enzymatic assembly of the F ribozyme from 7 fragments applying the following reaction conditions: 50 µl volumes (10 mM $MgCl_2$, 50 mM CHES pH 9) with 0.5 µM 5' fluorescently (FITC) labelled primer, 5' 2-Methylimidazole activated RNA oligonucleotides (1.5 µM each), 50 µM each RNA template strand at 37°C for 24h.b) Quantification of band intensities, the values (% of combined intensities in one line) are the mean of three individual experiments. c) Primer (P RP) extension on template (T RP)in ice for 7d of 1) control F ribozyme and 2) ligated ($F_{1234567}$) variant.

Supporting Tables

Table S1. Table of DNA and RNA sequences. DNA sequences are coloured in grey and RNA sequences in black. IVT denotes RNA sequences generated by in vitro transcription. Primer binding sites in template sequences are underlined and 5' modifications (FITC: Fluorescein isothiocyanate, Bio: biotinylation, C18: 18-atom hexa-ethylene-glycol spacer, p: phosphorylation) are marked in red. Standard oligonucleotides for the CBT selection procedure are as described previously.[1]

Application	Name	Description	Sequence 5' → 3'
Primer/ templates	P HP	Primer HP	FITCGAAGGGCA
	T HP	Template HP	UAUUGUUUCUCUCUUUGACUUCUCUGCCCUUC
	T HP C	Complement T HP for PAGE	GAAGGGCAGAGAAGUCAAAGAGAG
	P RP	Primer RP	FITCCUGCCAACCG
	T RP	Template RP	CAAUGAAUCCACGCUUCGCACGGUUGGCAG
	T RP complement	Complement T RP for PAGE	CUGCCAACCGUGCGAAGCGUG
	P F	Primer for F ribozyme	GGACAACCAAAAAGACAAATCTGCCC
	P F rev	Reverse primer for F ribozyme	GGATTATGACCTGGGCGTGTC
	P T7	Primer for fill-in	GATCGAGATCTCGATCCCGCGAAATTAATACGACTCACTATA
	F1a	Template 1 encoding the catalytic core	UUGGUUGUCCCGGUUGGCAG
	F1b	Template 2 encoding the catalytic core	AUUUGUCUUUCGGUUGGCAG
	F2a	Template 3 encoding the catalytic core	CUGAGGGCAGCGGUUGGCAG
	F2b	Template 4 encoding the catalytic core	UUCUCAAGCUCGGUUGGCAG
	F3a	Template 5 encoding the catalytic core	UCCGAAGAUGCGGUUGGCAG

	P pU	Primer for poly U extension	FITCUUUUUGACGUCUGCCAACCG
	T pU	Template for poly U extension, IVT	GGAAAAAAAAAAAACGGUUGGCAGACGUC
CBT selection	P RP CBT	Primer RP for CBT selection	BioUUUUUUUUUUCUGCCAACCG
	T RP CBT	Template RP for CBT selection	GUCAAUGACACGCUUCGCACGGUUGGCAGAAAAAAAAAA
	P HP CBT	Primer HP for CBT selection	BioUUUUUUUUUUGCGGUGUGAAGGGCA
	T HP CBT	Template HP for CBT selection	CUCUCUUUGACUUCUC UGCCCUUCACACCGC
	DC -3 RP	DNA minicircle -3 for T RP CBT	GTTACTTTTCAATGAATCCACGCTTCTGTAACGACTTTTCGGA<br><br>TTTCTAGGATCTCCAAGTATGTTCTAAGTC
	DC 0 RP	DNA minicircle 0 for T RP CBT	GTTACTTTTCAATGAATCCACGCTTCGCATGTAACGACTTTTC GGATTTCTAGGATCTCCAAGTATGTTCTAAGTC
	DC +7 RP	DNA minicircle +7 for T RP CBT	GTTACCTTTCAATGAATCCACGCTTCGCACGGTTGGTGTAAC GACTTTTCGGATTTCTAGGATCTCCAAGTATGTTCTAAGTC
	DC -4 HP	DNA minicircle -4 for T HP CBT	GTTACTTTTCATCTCTCTTTGACTTTCTTTGTAACGACTTTTCT GGATTTCTAGGATCTCGTAGTATGTTCTAAGTC
	DC 0 HP	DNA minicircle 0 for T HP CBT	GTTACTTTTCATCTCTCTTTGACTTCTCTTCTTTGTAACGACTT TTCTGGATTTCTAGGATCTCGTAGTATGTTCTAAGTC
	DC +4 HP	DNA minicircle +4 for T HP CBT	GTTACTTTTCATCTCTCTTTGACTTCTCTGCCTTCTTTGTAACG A CTTTTCTGGATTTCTAGGATCTCGTAGTATGTTCTAAGTC
	DC +6 HP	DNA minicircle +6 for T HP CBT	GTTACTTTTAATCTCTCTTTGACTTCTCTGCCCTTTCTTTGTAA CGACTTTTCTGGATTTCTAGGATCTCGTAGTATGTTCTAAGTC
Template for oligo-nucleotide ligation	F I	Piece 1 for templated ligation (4 pieces)	FITCC$_{18}$C$_{18}$GGACAACCAAAAAGACAAAUCUGCCCUCAGAGCU UGA
	F II	Piece 2 for templated ligation (4 pieces)	pGAACAUCUUCGGAUGUAGAGGAGGCAGCCUUCGGUGGC
	F III	Piece 3 for templated ligation (4 pieces)	pGCAAUAGCGCCAACGUUCUCAACAGAUACUUGACCUGAC

	F IV	Piece 4 for templated ligation (4 pieces)	pGAAAAGGCGCUGUUAGACACGCCCAGGUCAUAAUCC
	Sp I	Splint 1 (F I+F II) for templated ligation (4 pieces)	CCGAAGATGTTCTCAAGCTCTGAG
	Sp II	Splint 2 (F II+F III) for templated ligation (4 pieces)	TGGCGCTATTGCGCCACCGAAGGC
	Sp III	Splint 3 (F III+F IV) for templated ligation (4 pieces)	CAGCGCCTTTTCGTCAGGTCAAGT
	F 1	Piece 1 for templated ligation (7 pieces)	FITCC$_{18}$ C$_{18}$GGACAACCAAAAAGACAAAU
	F 2	Piece 2 for templated ligation (7 pieces)	pCUGCCCUCAGAGCUUGAGAA
	F 3	Piece 3 for templated ligation (7 pieces)	pCAUCUUCGGAUGUAGAGGAG
	F 4	Piece 4 for templated ligation (7 pieces)	pGCAGCCUUCGGUGGCGCAAU
	F 5	Piece 5 for templated ligation (7 pieces)	pAGCGCCAACGUUCUCAACAG
	F 6	Piece 6 for templated ligation (7 pieces)	pAUACUUGACCUGACGAAAAG
	F 7	Piece 7 for templated ligation (7 pieces)	pGCGCUGUUAGACACGCCCAGGUCAUAAUCC
	Sp 1	Splint 1 (F1+F2) for templated ligation (7 pieces)	CUGAGGGCAGAUUUGUCUUUUUGGUUGUCC
	Sp 2	Splint 2 (F2+F3) for templated ligation (7 pieces)	UCCGAAGAUGUUCUCAAGCU
	Sp 3	Splint 3 (F3+F4) for templated ligation (7 pieces)	CGAAGGCUGCCUCCUCUACA
	Sp 4	Splint 4 (F4+F5) for templated ligation (7	CGUUGGCGCUAUUGCGCCAC

		pieces)	
	Sp 5	Splint 5 (F5+F6) for templated ligation (7 pieces)	GGUCAAGUAUCUGUUGAGAA
	Sp 6	Splint 6 (F6+F7) for templated ligation (7 pieces)	CUAACAGCGCCUUUUCGUCA
Ribozymes	Z/Y/W library	IVT	GGACAACCAAAAAGACAAAUCUGCCCUCAGAGCUUGAGAAC AUCUUCGGAUGYAGAGGAGGCAGCCUUCGGUGGCGCRAUA GCGCCAACGUUCUCAACAGAYRCMCAA (N)$_{12,\ 18,\ 24}$ GACCUGA CGAAAAGGCGMUGUUAGACACGCCMAGGUCAUAAUCCCCG GAGCUUCGGCUCC
	Z	IVT	GGACAACCAAAAAGACAAAUCUGCCCUCAGAGCUUGAGAAC AUCUUCGGAUGCAGAGGAGGCAGCCUUCGGUGGCGCGAUA GCGCCAACGUUCUCAACAGACACCCAAUACUCCCGCUUCGG CGGGUGGGGAUAACACCUGACGAAAAGGCGAUGUUAGACA CGCCCAGGUCAUAAUCCCCGGAGCUUCGGCUCC
	K	IVT	GGACAACCAAAAAGACAAAUCUGCCCUCAGAGCUUGAGAAC AUCUUCGGAUGUAGAGGAGGCAGCCUUCGGUGGCGCAAUA GCGCCAACGUUCUCAACAGAUACCCAAAGACUACAACUUGA CCUGACGAAAAGGCGCUGUUAGACACGCCCAGGUCAUAAUC CCCGGAGCUUCGGCUCC
	C1	IVT	GGACAACCAAAAAGACAAAUCUGCCCUCAGAGCUUGAGAAC AUCUUCGGAUGUAGAGGAGGCAGCCUUCGGUGGCGCAAUA GCGCCAACGUUCUCAACAGAUACCCAAAGACUGAAACGUGA CCUGACGAAAAGGCGCUGUUAGACACGCCCAGGUCAUAAUC CCCGGAGCUUCGGCUCC
	C2	IVT	GGACAACCAAAAAGACAAAUCUGCCCUCAGAGCUUGAGAAC AUCUUCGGAUGUAGAGGAGGCAGCCUUCGGUGGCGCAAUA GCGCCAACGUUCUCAACAGAUACCCAAGGAAACUCUGAUGA CCUGACGAAAAGGCGCUGUUAGACACGCCCAGGUCAUAAUC CCCGGAGCUUCGGCUCC
	F	IVT	GGACAACCAAAAAGACAAAUCUGCCCUCAGAGCUUGAGAAC AUCUUCGGAUGUAGAGGAGGCAGCCUUCGGUGGCGCAAUA GCGCCAACGUUCUCAACAGAUACUUGACCUGACGAAAAGGC GCUGUUAGACACGCCCAGGUCAUAAUCC
Illumina sequencing	CL	cloning linker	pCTGTAGGCACCATCAAT/3ddC/
	P CL rev	Rev Primer for cloning linker	ATTGATGGTGCCTACAG
	P CL RP	RP Primer for cloning linker	GTACTGCTGCCAACCG

	P Seq	Primer for Illumina sequencing	AATGATACGGCGACCACCGAGATCTACACTCTTTCCCTACACGACGCTCTTCCGATCTNNNGAGTGGGTACTGCTGCCAACCG
	P Seqrev	Rev primer for Illumina sequencing	CAAGCAGAAGACGGCATACGAGATCGGTCTCGGCATTCCTGCTGAACCGCTCTTCCGATCTATTGATGGTGCCTACAG

Supplemental References

[1] A. Wochner, J. Attwater, A. Coulson, P. Holliger, *Science* **2011**, *332*, 209-212.

[2] J. Attwater, A. Wochner, P. Holliger, *Nat Chem* **2013**, *5*, 1011-1018.
